# Diffraction before destruction

**DOI:** 10.1098/rstb.2013.0313

**Published:** 2014-07-17

**Authors:** Henry N. Chapman, Carl Caleman, Nicusor Timneanu

**Affiliations:** 1 Center for Free-Electron Laser Science, DESY, Notkestrasse 85, 22607 Hamburg, Germany; 2 Department of Physics, University of Hamburg, Luruper Chaussee 149, 22761 Hamburg, Germany; 3 Centre for Ultrafast Imaging, Luruper Chaussee 149, 22761 Hamburg, Germany; 4 Department of Physics and Astronomy, Uppsala University, Box 516, 75120 Uppsala, Sweden; 5 Laboratory of Molecular Biophysics, Department of Cell and Molecular Biology, Uppsala University, Box 596, 75124 Uppsala, Sweden

**Keywords:** protein crystallography, radiation damage, X-ray lasers

## Abstract

X-ray free-electron lasers have opened up the possibility of structure determination of protein crystals at room temperature, free of radiation damage. The femtosecond-duration pulses of these sources enable diffraction signals to be collected from samples at doses of 1000 MGy or higher. The sample is vaporized by the intense pulse, but not before the scattering that gives rise to the diffraction pattern takes place. Consequently, only a single flash diffraction pattern can be recorded from a crystal, giving rise to the method of serial crystallography where tens of thousands of patterns are collected from individual crystals that flow across the beam and the patterns are indexed and aggregated into a set of structure factors. The high-dose tolerance and the many-crystal averaging approach allow data to be collected from much smaller crystals than have been examined at synchrotron radiation facilities, even from radiation-sensitive samples. Here, we review the interaction of intense femtosecond X-ray pulses with materials and discuss the implications for structure determination. We identify various dose regimes and conclude that the strongest achievable signals for a given sample are attained at the highest possible dose rates, from highest possible pulse intensities.

## 1. Introduction

The determination of the structure of macromolecules at the atomic scale must contend with the effect of radiation damage. To resolve features at this length scale requires radiation of comparable wavelength or shorter, which, for X-rays or electrons, will be energetic enough to ionize atoms and break bonds, leading to changes in the very structure under investigation [1]. Any measurement of the scattered radiation from a sample, such as X-ray diffraction from a protein crystal, requires an adequate exposure for that recording. The energy transfer into the sample during that exposure depends on the atomic cross sections for photoabsorption relative to those for elastic scattering. The ratio of scattering to absorption cross sections can be varied only by a change in wavelength, and there is no way to avoid the fact that for every scattered X-ray photon there are many more photons absorbed by the sample. Up until recently, the most successful way to work within this fundamental constraint has been to grow large well-diffracting crystals that yield strong diffraction within the damage-limited exposure. Cryogenic cooling of the sample reduces some of the reactions that occur subsequently to photoabsorption allowing for about a 30–50 times increase in exposure [2–4]. A radical new approach to overcome the effects of radiation damage has recently been demonstrated [5,6], using femtosecond flashes of X-rays that are shorter than the time it takes atoms to move [7]. Doses that are thousands of times higher than conventional limits have been achieved, which allows for a corresponding reduction in crystal volume and measurable diffraction from crystallites consisting of single mosaic blocks.

Flash X-ray crystallography, such as flash photography, freezes motion of the sample and can be used to capture snapshots of fast events. If no component of the sample moves faster than the speed given by the ratio of the spatial resolution to the pulse duration, then the structural information recorded will be free of artefact owing to blurring [8]. This concept extends all the way to the atomic scale, using femtosecond pulses from X-ray free-electron lasers (FELs). The large increase in tolerable dose that can be achieved means that diffraction of high signal to background can be recorded from samples with a corresponding decrease in volume, which has opened up the new method of protein serial nanocrystallography [5] to determine structures from snapshot diffraction patterns recorded from a stream of crystals of submicrometre diameter. The X-ray pulses are necessarily intense, containing about 10^12^ photons in tens of femtoseconds duration. When focused onto the sample, intensities can far exceed 10^17^ W cm^−2^ which completely vaporizes the sample, but the full destruction occurs after the pulse has passed through the sample. Under these conditions, there is no requirement to cool the sample, and measurements can be made at room temperature.

With repetition rates of 120 pulses per second at the Linac Coherent Light Source (LCLS) [9], and capable detectors with corresponding frame rates [10], a natural approach to data collection is by recording serial snapshots from a flowing liquid suspension of protein crystallites. This method of serial femtosecond crystallography (SFX) was first carried out at the LCLS in December 2009, at a long wavelength of 6 Å (2 keV photon energy) on photosystem I and lysozyme crystals [5,11,12]. Doses above 1 GGy were achieved. The method was soon extended to shorter wavelengths when the appropriate instrumentation and beamline became available [6,13]. As practised so far, the method uses a gas-focused liquid jet of the crystal suspension that flows across the focus of the X-ray beam. The jet diameter can range from 500 nm diameter to several micrometres. This narrow diameter gives a low background, so that sufficient signal to background can be achieved from submicrometre crystals. Detector frames are collected on every X-ray pulse while the suspension flows continuously. A crystal of unknown and random orientation is hit by chance, depending on the concentration of crystals in the suspension, and subsequent analysis of all detector frames finds the crystal hits from the misses.

At 120 frames per second, over 400 000 frames are collected per hour, and typical measurements run several hours to obtain tens or hundreds of thousands of single-crystal diffraction patterns. Each crystal obviously does not rotate significantly during its flash exposure and so measured Bragg peaks are not necessarily fully integrated. By averaging the counts of indexed Bragg spots, we obtain an accurate set of three-dimensional structure factors [14]. This dataset, aggregated from the many single-crystal patterns, can be thought of as a three-dimensional powder diffraction pattern. The difference between this and the usual two-dimensional powder pattern obtained by summing all single-crystal patterns is simply the order of operations: indexing followed by summing compared with summing and then indexing in two-dimensional powder diffraction. The integration over crystal shapes, orientations and quality is the same in both cases with the advantage for SFX data analysis of being able to filter out poor-quality crystals and discarding the misses that would only contribute to background. Both two- and three-dimensional powder diffraction have the advantage over single-crystal diffraction that the exposure of each crystal can be much lower than required to achieve a measurable signal above background in Bragg spots. This gives a second and distinct strategy in which serial crystallography overcomes the dose-limited resolution of small crystals. As in cryoelectron microscopy [15], any non-systematic background and noise can be averaged away with enough measurements, even if the signal cannot be discerned in a single pattern (i.e. the signal to background may be much less than unity). The signal requirement in a single pattern is not to achieve detectable signal to background at a particular resolution but only to achieve enough signal to be able to determine the orientation of the crystal, so that the data can be merged in three dimensions [16]. This requirement may reduce the necessary achievable signal by orders of magnitude, especially if the stronger low-resolution peaks can be used for indexing, but comes at a cost of requiring many more patterns. This approach should allow even smaller crystals than have even been used to date, ultimately down to the single molecule [7].

In addition to software that acquires and assembles the data from the detector, two suites of software have been developed to treat the extremely large volume of data collected in the serial method. The first processing is carried out by Cheetah [17] which detects crystal hits and carries out background subtraction. The CrystFEL suite [18,19] carries out the indexing and predictive location of peaks, and Monte Carlo integration to give the structure factors that can be assessed and phased using standard crystallographic software. Several structures have been solved by this technique [20–23].

For all structures solved so far, no evidence of radiation-induced atomic displacement has been observed, even in the vicinity of heavy atoms which could impart forces on surrounding atoms that are higher than average [24]. When compared with synchrotron structures, the FEL structures appear to have much better definition of side chain densities, salt bridges and disulfide bonds [23,25]. Where conformational differences exist, these may be due to differences in temperature (room temperature versus cryogenically cooled) or owing to radiation damage in the synchrotron sample [23]. Most structures have been obtained with relatively moderate dose of 10–200 MGy, far below expected tolerable limits. The dose in these experiments has often been restricted to avoid damaging the detector with strong low-angle Bragg diffraction. At higher intensities (or doses), it is expected that atomic scattering factors of heavier elements will ‘bleach’ owing to excessive ionization, providing a convenient method for phasing [26,27]. This high-dose regime has not been fully explored, and it appears that we are still far from experiencing ultimate dose limits, even with the continued development of X-ray sources. For example, with pulse durations of 1 fs and lower, it will be possible to outrun Auger decay, creating hollow atoms that frustrate further photoabsorption [28,29].

Here, we present an overview of the interaction of intense femtosecond X-ray pulses with materials and discuss the implications for structure determination. We carry out calculations based on atomic cross sections which give indications of the processes occurring during X-ray FEL exposures and regimes for exploiting high-intensity phasing. Similarly, we determine the low-dose regime, where photons that are elastically scattered are likely to do so from atoms that have not yet experienced any interaction with X-rays or ejected electrons. We review simulations, based on molecular dynamics and plasma dynamics codes, that predict the evolution of matter in the intense X-ray pulse and the effect on the diffraction pattern, along with some supporting experiments. There is still much to study and understand, and hopefully more experiments will shed light on the opportunities of ‘diffraction before destruction’.

## 2. Dose

Dose is defined as the energy deposited in the sample per unit mass, and the degree of radiation damage depends upon this quantity, because it quantifies the number of primary inelastic interactions per atom or molecule. The SI units for dose are Gray, with 1 Gy = 1 J kg^−1^. For protein of average composition of H_50_C_30_N_9_O_10_S_1_ and density 1.35 g cm*^−^*^3^ [3], 1 MGy corresponds to 0.076 eV per atom, and in water, 1 MGy equates to 0.062 eV per atom. In the regime of kinematical diffraction, or single scattering, the dose depends on the probability that an atom absorbs a photon multiplied by the energy of that photon, which is given by the atomic cross section *σ_A_* multiplied by the X-ray fluence *I*_0_ (the pulse energy per unit area, which is equal to the number of photons per unit area times the photon energy, or 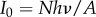, where *N* is the number of photons, *h* Planck's constant, *ν* frequency and *A* the beam area). Because *N_A_* atoms have a mass of *m_A_*, the energy deposited per unit mass, rather than per atom, of material of a single atomic constituent is then2.1
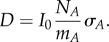
Here, we assume that samples that are much thinner than their absorption length (the thickness for a transmission of 1/*e*), so that we can ignore the absorption by the part of the sample facing the incoming X-rays or indeed the geometry of the sample. Under this premise, the dose is an atomic property, not dependent on the arrangement of atoms except as to how many atoms are presented per unit area to the X-ray beam through the thickness of the sample.

The photoabsorption cross sections *σ_A_* for the elements have been tabulated by Henke *et al.* [30] and can be accessed, along with scattering cross sections and optical constants in the online ‘Henke tables’ maintained by Eric Gullikson of the Lawrence Berkeley National Laboratory Center for X-ray Optics website (http://henke.lbl.gov/optical_constants/). Cross sections are also available in the International Tables of Crystallography Section 4.2.4 [31]. The cross sections of various charge states of ionized atoms that may be formed by a high-fluence X-ray beam can be calculated using the XATOM toolkit [32]. At a photon energy of 6 keV, atomic absorption cross sections are of the order of 100 barn for light elements to 100 kbarn for heavier elements, or 10*^−^*^6^ to 10*^−^*^3^ Å^2^, much smaller than the ≈0.9 Å^2^ area of a hydrogen atom taken as a circle of the Bohr radius. This, of course, results in the high penetration of X-rays through matter. The number of photons per unit area needed to ensure that any particular atom will absorb a photon is given by 1/*σ_A_*. The absorption cross section of carbon for a 6 keV photon is 2.2 × 10*^−^*^6^ Å^2^ which means that 4.5 × 10^5^ photons/Å^2^ = 4.5 × 10^13^ photons μm^−2^ will photoionize every carbon atom in the sample. In such a case, the product of the cross section and the number of photons per unit area is 1, and so *I*_0_*σ_A_* = *hν*, the photon energy. The one-photon-per-atom absorption dose is then given by2.2
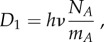
and increases linearly with photon energy, because this is the energy delivered per interaction. For a pure-carbon sample, the one-photon-per-atom absorption dose at *hν* = 6 keV is 48 GGy, over 1000 times higher than the 30 MGy tolerable dose for slow exposures of cryocooled macromolecules [2]. Said another way, less than 0.06% of carbon atoms are photoionized at a dose of 30 MGy, even though this is about the highest tolerable dose for slow exposures of cryogenically cooled samples. From equation (2.2), heavier elements have lower one-photon-per-atom absorption doses: this dose for a pure Mn sample, for example, is 10 GGy at 6 keV.

For a sample of any particular stoichiometry, the dose can be calculated by averaging over the atomic cross sections of the constituents weighted by their relative abundances. Because the measurable effect of photoabsorption is the loss of transmission through a material, the weighted average cross sections can conveniently be expressed in terms of the absorption coefficient *μ* of the material, where the transmission through thickness *t* of the sample is *I* = *I*_0_exp(*−μt*). Because *N_A_*/*m_A_σ_A_* = *μ*/*ρ*, we can thus express dose as2.3
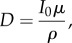
for a sample density *ρ*.

As an example consider an average protein of empirical formula H_50_C_30_N_9_O_10_S_1_ and density 1.35 g cm*^−^*^3^. At a photon energy of 6 keV and 10^12^ photons μm*^−^*^2^, the pulse fluence is *I*_0_ = 96 kJ cm*^−^*^2^, and the absorption coefficient is 32.5 cm*^−^*^1^ giving a dose of 2.3 GGy. Note that a 1 μm thick protein crystal is much thinner than the absorption length of 1/*μ* = 308 μm. A plot of dose versus photon energy for a given incident fluence (in terms of photons per square micrometre) is given in fig. 1 of Howells *et al.* [3]. X-ray FEL pulses are generated with approximately constant pulse energy across the tuning range of the FEL, giving a maximum photon number inversely proportional to the photon energy. A more comprehensive and accurate calculation of dose, taking into account the sample geometry and environment, can be carried out using the RADDOSE program [33].

**Figure 1.**
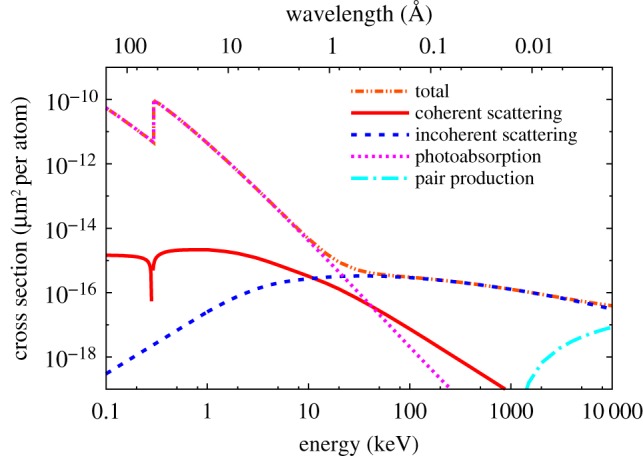
Atomic cross sections of neutral carbon for photoabsorption, elastic scattering, and inelastic (Compton) scattering. The carbon *K*-shell absorption edge is visible at 284 eV photon energy. The cross sections are plotted in the unusual units of µm^2^ per atom, because this shows the inverse of how many photons are required per square micrometre to photoionize or scatter from any atom in the beam. (1 barn = 10^−24^ cm^2^ = 10^−16^ μm^2^). (Online version in colour.)

Regarding our example of the saturation of Mn given above, the fluence to achieve saturation of Mn absorption is *I*_0_ = *hν*/*σ_A_* for Mn, which is 140 kJ cm*^−^*^2^ at *hν* = 6 keV. Thus, for a dilute fraction of Mn in our average protein, the Mnone-photon photoionization saturation is achieved at a dose to the protein sample of 3.4 GGy at 6 keV (below the Mn *K*-edge) and 520 MGy at 8 keV (above the Mn *K*-edge). The doses to a protein sample for which every atom of a particular element in that sample have absorbed a single photon are given in table 1.

**Table 1.** Doses to achieve one photoionization per atom for various elements, and their corresponding dose rates to produce hollow atoms. The dose is calculated as that received by a sample of H_50_C_30_N_9_O_10_S_1_ of 1.35 g cm*^−^*^3^ at the one-photon-per-atom absorption fluence of the particular element. The Auger decay rates are computed from lifetimes given in [34].

element	*D*_1_ at 6 keV (GGy)	*D*_1_ at 8 keV (GGy)	Auger decay time (fs)	hollow atom dose rate at 6 keV (GGy fs^−1^)	hollow atom dose rate at 8 keV (GGy fs^−1^)
C	103	144	10	10.30	14.40
N	53.4	74.7	7.1	7.52	10.52
O	30.5	43.0	5.0	6.10	8.60
S	2.01	2.60	1.3	1.55	2.00
Mn	3.36	0.52	0.62	5.42	0.84
Fe	2.86	0.46	0.55	5.20	0.84

The diffraction signal is dependent on the atomic coherent scattering cross sections of atoms. In the X-ray range, the scattering cross section is usually much smaller than the absorption cross section. For carbon, the scattering cross section *σ_S_* is 6 × 10*^−^*^16^ μm^2^, and so for every scattered photon, there are 30 photoionization events. The cross sections for carbon are plotted in figure 1, showing a stronger dependence on photon energy for the absorption cross section than coherent (elastic) scattering. But note that the diffraction signal also depends on the scattering geometry which results in an additional *λ*^3^ dependence owing to the detectors intersection of *q* space and the width of Bragg peaks (see §5).

## 3. Damage processes

The dose described above quantifies the transfer of energy from the X-ray beam into the sample. The effect of this on the sample, and hence on the recorded diffraction pattern, depends on the evolution of the processes initiated by the primary photoionizing interaction. These processes crucially depend on the exposure time, as emphasized in this paper, but also on the escape of energy from the sample, for example, through photoelectrons that are completely ejected from the surface. If we consider long enough time scales where the sample reaches a thermal equilibrium, then, if no energy flows out of the sample (including no escaping photoelectrons), the temperature rise is given by the dose divided the heat capacity of the sample. The heat capacity of water, for example, is 4800 J kg K^−1^, so 1 MGy dose would heat water by 10^6^ J kg/(4800 J kg K^−1^) = 208 K. This does not depend on sample size, because both dose and heat capacity are intrinsic properties of matter. In slow synchrotron exposures, there is ample time for this heat to be conducted away to unexposed parts of the sample and the environment, unless the sample is well insulated, so it is unlikely that such temperature rises occur in protein crystallography experiments. Nevertheless, this calculation nicely illustrates how much energy the X-ray beam can deliver and gives an intuitive way to contemplate dose. An X-ray FEL pulse delivering a 1 GGy dose can indeed heat the sample up to 200 000 K, creating a plasma that cools by expansion (long after the pulse). Plasmas are created at even lower doses. Another intrinsic scale to consider is the energy required to break all bonds in the sample. The binding energy of a carbon–carbon bond is about 1 eV, or 100 kJ mol^−1^. For a pure-carbon sample, this corresponds to a dose of 100 kJ mol^−1^/(12 g mol^−1^) = 8 MGy.

**Figure 2.**
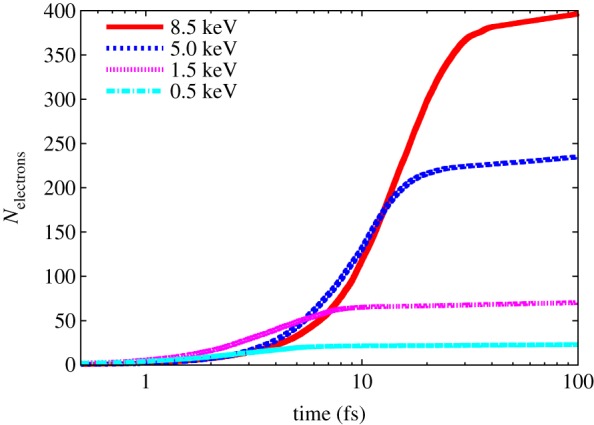
The cascade of electrons generated by a single electron of energy 500, 1.5, 5 and 8 keV in a urea crystal (CON_2_H_4_) as calculated by molecular dynamics code [40,41]. The number of secondary electrons generated is plotted as a function of time after the photon absorption event. After 100 fs, there is an electron generated per 21 eV of absorbed energy. The majority of secondary electrons are created in less than 20 fs. (Online version in colour.)

The photon absorbed by an atom instantaneously ejects a photoelectron with an energy given by the photon energy minus the binding energy. For light elements such as C, N and O, these photoelectrons will have quite high energy. For example, for a 6 keV photon, the *K*-shell photoelectron from C will have an energy of 5716 eV. This electron is not relativistic but its speed is still rather high at 450 Å fs^−1^. For light elements, the ions relax predominantly by Auger decay, a radiationless decay that releases an Auger electron of a fixed energy from the valence shell as another valence electron fills the core shell hole. For C, the energy of the emitted Auger electron is about 230–280 eV [35] with an initial speed of 100 Å fs^−1^. These free photoelectrons and Auger electrons propagate through the sample and collide with other atoms of the sample. The collisions can ionize those atoms, and the initial electron energies (especially those of the photoelectrons) can be high enough even to eject *K*-shell electrons. The mean-free path of the 5.7 keV photoelectrons is about 150 Å, but quickly drops as the electron loses energy through the collisions, generating a cascade of electrons that eventually thermalize. This cascade has been modelled for electrons of different initial energies in carbon [36,37] and other light elements [38–41] and it is found that the cascade generated by a single 5 keV photoelectron thermalizes in about 10 fs and produces around 240 ionizations, 10 of which are core–shell ionizations. The calculations are in agreement with recent measurements at the LCLS where a solid-density plasma was created by focusing an X-ray FEL pulse onto an Al foil, and studied by observation of *K*-shell fluorescence [42]. The measured fluorescence spectra revealed that ionization was indeed dominated by electron-ion collisions. The theoretical work of Ziaja *et al*. considers electron capture (i.e. neutralization of atoms by free electrons) but finds this to be rather insignificant over the 100 fs time durations of the cascade. The free electrons do affect the binding energies of bound electrons (known as continuum lowering) and so ionization rates depend on the plasma environment. The range of the electron cascade is about 100 nm and is centred around the primary ionization. A plot of the number of electrons generated per primary ionization in a urea crystal is shown in figure 2, for various initial electron energies. It is seen that the photoelectrons create more collisional electrons than do the lower-energy Auger electrons.

We therefore see that the cascade of electrons creates vastly more ionizations than the X-rays themselves, with each 6 keV photoelectron leading to a region of about 285 ionized atoms in a volume of about 50 nm radius. Higher photon energies lead to more ionizations over a larger volume, with almost double the diameter and number of ionizations for a 12 keV photoelectron compared with 6 keV. On average, it takes about 21 eV of photon energy per ionization. The volume of 50 nm radius contains about 107 atoms, but consider a crystal of the average protein listed above of 1 μm^3^ volume, irradiated with a 1 MGy dose. From equation (2.3), given *μ* = 32.5 cm*^−^*^1^ at 6 keV photon energy, this dose is achieved with 41 J cm^−1^ or 4 × 10^8^ photons μm*^−^*^2^. In the 1 μm^3^ crystal, there are about 10^11^ atoms, and from the atomic cross sections multiplied by the number of atoms, we can easily estimate that there will be 1.2 × 10^6^ photoionizations (2.7 × 10^5^ from C, 1.5 × 10^5^ from N, 3.0 × 10^5^ from O and 4.6 × 10^5^ from S) for 6 keV photon energy. At this dose and higher, therefore, there are more photoabsorptions than number of 50 nm radius regions in the crystal, and so we can consider all ionizations to occur randomly and isotropically throughout the crystal volume. In total, there will be about 2.5 × 10^8^ ionization events out of a possible 10^11^ atoms, and about 50 000 scattered photons. For a protein of 300 residues (5760 atoms), this corresponds to 19 ionizations per molecule and only one scattered photon per 350 molecules. A dose of 20 MGy gives one ion per residue. For exposure times that are considerably shorter than the duration of the cascade, there will be obviously less ionized atoms during the exposure, as can be estimated from figure 2, and with pulses of 1 fs duration or less the cascades can almost completely be ignored. Such exposure times will also outrun Auger decay times of the lighter elements, so that scattering will be either from neutral atoms or singly ionized (core–hole) atoms.

For X-ray FEL exposure times ranging from about 1 fs to 100 fs, the structural changes that can affect the diffraction pattern are the ionization of atoms, which changes the atomic scattering factors, and the displacements of atoms owing to Coulomb forces on those ions. As seen from the analysis above, it takes a considerably higher dose than 1 MGy for every atom to be ionized during exposures of about 100 fs. The dose at which the number of free electrons equals the number of atoms by the end of the exposure is about 400 MGy for the ‘average protein’. At this dose, photons will still predominantly scatter from pristine atoms that have neither been photoionized or collisionally ionized, because the pulse-averaged ionization at this dose will be 0.5 electrons per atom. This is calculated taking a very simple model of an exposure time longer than the time it takes a cascade to develop and a flat-top pulse (i.e. a constant rate of photoionizations during the exposure). At doses less than this one-electron-average-ionization dose of 400 MGy, X-ray scattering will predominantly be from neutral (undamaged) atoms. This is of particular relevance for the structure determination of metalloproteins where the redox-active centres will be unlikely to be modified (either oxidized by ionization or reduced by electron capture or transfer) at this dose or lower with short pulses, and for anomalous diffraction as was nicely demonstrated with FEL pulses by Barends *et al*. [43] (at a dose of 22 MGy). All other atoms that contribute to the diffraction pattern will be similarly likely to be pristine and free of damage. It also sets the limit when electron or fluorescence spectroscopy can be carried out without significant artefact, as was well satisfied in the fluorescence spectroscopy of Mn atoms of photosystem II by Kern *et al*. [44].

The presence of heavy atoms does lower the one-electron-average-ionization dose limit owing to the higher cross sections of those atoms. The energies of photoelectrons emitted from these atoms will be considerably lower than from light elements, depending on how far the photon energy is above the absorption edge and the Auger electrons will generally be higher energy but compete with fluorescence. These elements do have higher electron inelastic scattering cross sections and so may be more likely to be ionized than the lighter elements. As mentioned above, the same dose from an X-ray pulse that is considerably shorter than the cascade time of 100 fs will experience less ionization during the exposure. A given pulse fluence *I_0_* delivered with a shorter pulse duration *T* means the pulse intensity *I*_0_/*T* is higher. As is discussed further below, higher intensity is also beneficial from the point of view of outrunning X-ray-induced atomic motion. Higher intensity requires a source that produces more photons per unit time, that is higher peak power. From equations (2.1) and (2.3), we see that dose rate is proportional to pulse intensity.

**Figure 3.**
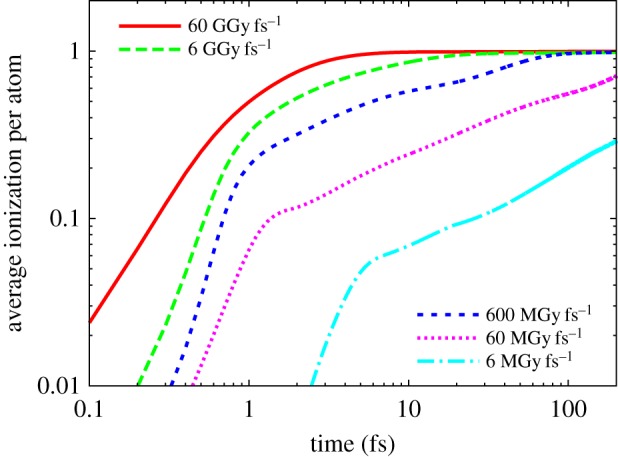
The evolution of the average ionization of atoms in a sample illuminated by an X-ray pulse at various dose rates, as calculated by plasma dynamics code. The ionization per atom is normalized to 1, corresponding to full ionization in all atoms. (Online version in colour.)

Although the photoelectron energies depend on photon energy, as do the number of electrons generated in a cascade, it is found in simulations that the electrons quickly thermalize and that the distribution of electron energies produced, for a given amount of energy instantaneously absorbed per atom in the sample (i.e. for a given instantaneous dose), is largely independent of photon energy [45]. As a consequence, the evolution of the degree of ionization and atomic displacements during the pulse, for a given dose rate, is independent of photon energy to a good approximation. This approximation is best for samples consisting of light elements. A plot of the average ionization of non-hydrogen atoms as a function of time *t* during the pulse is given in figure 3 for various dose rates for flat-top pulses (i.e. pulses of constant intensity), as computed by a plasma dynamics code [46] for a protein of general composition of H_141 400_O_57 300_C_16 900_N_3310_S_89_Fe_12_Mg_96_P_3_Ca, corresponding to the average composition of a photosystem I crystal containing 78% water as solvent and a density of 1.077 g cm*^−^*^3^.

We note that the electron cascade can be vastly reduced if the photoelectrons can escape the sample, which will occur for isolated samples that are smaller than the mean-free path of the photoelectrons. This will vastly reduce the damage processes that occur in the sample, at least for a short time until the photoelectrons start to become trapped in the potential of the highly charged object. Once the average ionization reaches unity in a 8 nm diameter spherical protein particle, 6 keV electrons can no longer escape that particle [47]. Similarly, if the X-ray beam radius is smaller than the electron cascade radius, then the energy is spread over more atoms than those doing the scattering, reducing the damage processes occurring in the illuminated volume. Again, space charge will limit the time for which electrons can escape that volume.

### (a) High-intensity pulses

The one-photon-per-atom absorption dose for light elements is of course much higher than the one-electron-average ionization limit, dependent on the photoabsorption cross sections. If these doses are achieved within the Auger decay time of the atom, then it becomes probable to ionize the remaining core–shell electron, creating atoms with empty core shells. The dose rates required for this, estimated from the one-photon saturation dose divided by the Auger decay rate are given in table 1 for different elements. At these dose rates, and exposure times as short or shorter than the Auger decay times of several femtoseconds, photoabsorption becomes frustrated [28,29], because the hollow atom cannot effectively absorb a photon. Such exposure times are also shorter than the time for electron cascades to develop. Hollow atoms will continue to scatter photons, with a cross section (at low *q*) approximately proportional to the number of remaining electrons, so this is very favourable for diffraction measurements. Under these conditions where the population of excited ions exceeds ground state ions, the decay by fluorescence can stimulate emission in other excited ions, creating a *protein laser* and reducing the number of Auger electrons generated [48].

The dose required to remove all valence electrons depends on the pulse duration and if it is long enough for the electron cascade to fully develop. In that case, assuming 25 eV per valence ionization, collisional ionization would saturate at a dose of about 100 eV per atom, corresponding to 1.3 GGy. This is lower than the 100 GGy one-photon-per-atom dose for C, but comparable to the 2 GGy one-photon-per-atom dose for S (table 1). For 10 fs pulses or shorter, the cascade is not as influential. The complete removal of all electrons from the light elements is possible through multiple photoabsorption events per atom, possibly with relaxation processes between. This would require at least 1.5 keV of absorbed energy per atom in the ‘average protein’, or a dose of about 20 GGy. For pulses shorter than the Auger lifetimes, collisional ionization owing to Auger electrons will be avoided by there will still be a cascade of ionization owing to the photoelectrons. Son *et al*.'s [26] analysis shows that even these can be outrun with pulses that are shorter than the time the photoelectron makes its first impact, dependent on the speed of that electron and its mean-free path. For 12 keV photons, this requires 0.1 fs pulses [26] in a pure-carbon sample, allowing exposures of up to 10^15^ photons μm^−2^, corresponding to a dose rate of 2300 GGy fs*^−^*^1^.

### (b) Atomic motion in a plasma

**Figure 4.**
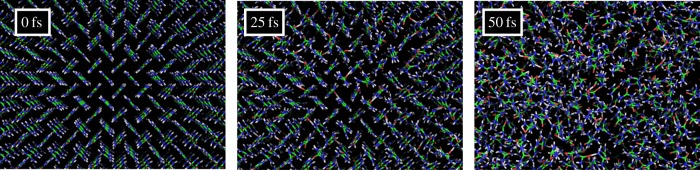
Molecular dynamics simulations of the evolution of a urea crystal exposed to a 50 fs X-ray pulse of 5 × 10^12^ 9 keV photons focused to 1 μm^2^. Views of the crystal are shown before illumination, after 25 fs, and after 50 fs. This intensity corresponds to a dose rate of 50 MGy fs*^−^*^1^. Reproduced from [46]. (Online version in colour.)

**Figure 5.**
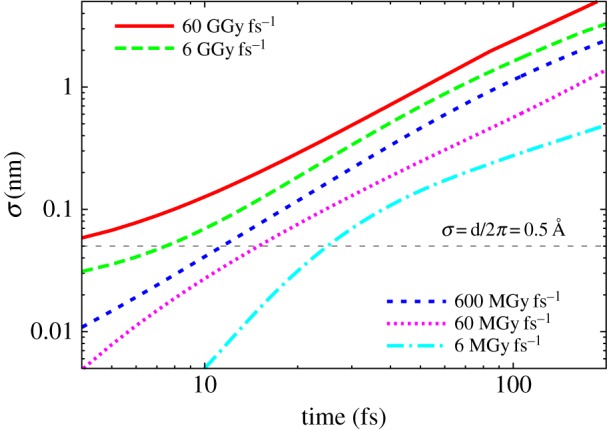
Plots of the RMS atomic displacement as a function of time during pulses at various dose rates, as calculated by plasma dynamics code in a sample matching the composition of photosystem I and which is much larger than the photoelectron range [46]. The curves are averaged over photon energies from 2 to 9 keV. (Online version in colour.)

The atomic motion induced by X-ray FEL pulses has been modelled by molecular dynamics [7,41] and plasma physics codes [46,49]. The latter approach treats the fast processes mentioned above such as three-body recombination and continuum lowering. The breaking of all carbon–carbon bonds at a dose of 8 MGy, predominantly through the ionizing and non-ionizing collision processes of the electron cascade, and the large number of ionizations, leads to the formation of a plasma when that dose is delivered by a short pulse. At dose rates of about 1 MGy fs*^−^*^1^ and higher, molecular dynamics simulations show random and isotropic displacements of all atoms that increase during the pulse, as shown in figure 4. The simulations are in good quantitative agreement with plasma physics simulations, valid in cases when all bonds are broken. In a plasma, the motion of ions can be described by a diffusion equation, determined from the ion velocities and the frequency of ion–ion collisions. The diffusion ‘constant’ 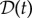 increases during the pulse as energy is continually deposited into the system and the root-mean square (RMS) ion displacement accelerates according to the diffusion equation3.1
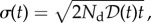
where *N*_d_ is the number of dimensions. Simulations for various photon energies show that 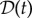 is approximately dependent on dose rate. That is, the evolution of the RMS displacement at a given intensity *I*_0_/*T* at 6 keV is approximately equal to that at twice the intensity at 8 keV. A plot of the RMS displacement as a function of time *t* during the pulse is given in figure 5 for various dose rates for flat-top pulses (i.e. pulses of constant intensity), as computed by the plasma code for protein [45]. It is found that the RMS displacement roughly increases with *t*^3/2^ and the dependence on intensity is as (*I*_0_/*T*)^1/2^.

Correlated motion of atoms could occur, even under the conditions of the formation of a dense plasma, because the initial undamaged structure is certainly inhomogeneous, and initial trajectories of atoms could be influenced by this initial structure. One influence on these initial trajectories is the presence of heavy elements. Jurek & Faigel [24] have predicted the repulsion of light elements by a rapidly ionizing heavy atom, for example. They performed molecular dynamics simulations on an isolated 40 Å diameter carbon particle with iron atoms embedded, at a dose of 226 GGy, and found that there was an expansion of the innermost shell of carbon atoms away from the iron atom they surrounded, and that this motion was faster than the RMS displacement of the rest of the carbon atoms. Another form of correlated motion could include migration of charge from light elements to a neighbouring rapidly ionizing heavy atom [50]. The influence of these motions on the diffraction pattern is discussed in §4.4.

### (c) Slow exposures

Here, we give a very superficial account of the damage processes that occur during the long exposures that are typical for protein crystallography at synchrotron sources, for comparison with the billion-times shorter FEL pulses. The electron cascade that follows each photoabsorption event will be the same as for FEL exposures, although another photoionization event in the 50 nm radius volume of the cascade is unlikely to happen for a time comparable to the exposure time, so the electron temperature does not reach such high values and a plasma does not develop. The energy delivered will certainly thermalize (and flow out of the sample into the environment), and some ions may be neutralized through electron capture. The processes of radiolysis that occur are complex and are predominantly initiated by the cascade of electrons that may end up as solvated electrons and hydroxyl radicals (which can be stable for nanoseconds, a million times longer than FEL pulses [51]). These radicals diffuse, at a rate that is temperature-dependent, and interact with reactive components of the sample, such as metal centres, disulfide bridges, decarboxylation of aspartate and glutamate residues, loss of the OH group from tyrosines and the scission of the C–S bond in methionines [52]. Davis *et al*. [53] provide a model of damage from such reactions. Fuller treatments than given here of the global and specific radiation damage of macromolecular crystals can be found in a growing literature (see for example [54] and references therein).

## 4. Scattering from exploding crystals

The diffraction pattern of a sample can be derived from the coherent sum of scattered waves from the constituent point scatterers (atoms), taking into account the path differences of those waves, or the Fourier transform of the electron density obtained on the Ewald sphere. The measured pattern is given by the square modulus of the wavefield4.1
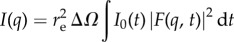
and4.2
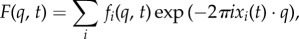
where *r*_e_ is the classical electron radius, Δ*Ω* is the solid angle of a detector pixel, *f_i_*(*q*, *t*) are the atomic scattering factors of atoms at locations *x_i_*(*t*), explicitly written here as dependent on time *t* during the pulse of intensity *I*_0_. The magnitude of the momentum vector *q* is 2sin*θ*/*λ*, where *θ* is the Bragg angle. The sum is over all atoms in the sample. The three-dimensional datasets obtained by nanocrystallography or single molecule diffraction combine many diffraction measurements incoherently, summing over variations in crystal shapes and sizes, as well as other fluctuating variables such as the pulse spectrum and energy. If every molecular unit in every particle or nanocrystal was exactly the same and not perturbed by the beam, and its orientation could be precisely determined, then, in the limit of many patterns, this dataset would be equivalent to the diffraction collected from a single undamaged particle or nanocrystal as it would be rotated through all necessary orientations. In real experiments, of course, the effect of the interaction of the pulse with the sample will cause disorder of the structure that will be different from unit cell to unit cell and particle to particle (or crystal), and it is this variability of the sample structure that leads to a degradation of the diffraction pattern, and potential loss of structural information. The structural variation can be caused by the displacement of atoms as well as a change in the scattering factors of atoms that occurs on ionization. As discussed above, photoionization occurs immediately, so while it is possible to overcome atomic displacements with fast enough pulses, it is only possible to outrun the bulk of collisional ionization with subfemtosecond pulses [26].

### (a) Atomic scattering factors

**Figure 6.**
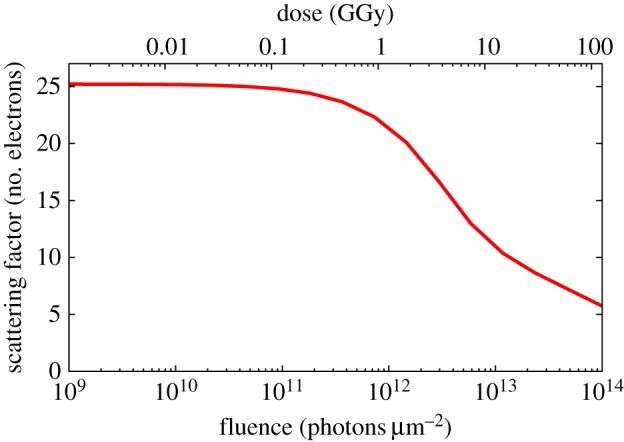
Magnitude of the effective form factor of Fe atoms in a protein sample as a function of pulse fluence (or equivalently, dose) for a photon energy of 8.1 keV (trial mode). Reproduced with permission from [27]. (Online version in colour.)

To first approximation and at low resolution, the scattering factor of an ionized atom is proportional to its number of bound electrons. The scattering factors of core–shell-ionized atoms (i.e. ‘hollow’ atoms) decrease at high resolution compared with a neutral atom, because charge is not as localized around the nucleus: the difference of the shape of the scattering factor is only significant above 1.7 Å resolution [32]. Near the absorption edge of an element, the atomic scattering factor varies considerably, and the complex-valued dispersion correction changes abruptly with photon energy. The position of the edge changes dramatically depending on the charge state of the atom, and the *K*-shell absorption edge of Fe, for example, can shift to higher energy by more than 1 keV for high charge states [26]. This is because the binding energy of the core electron becomes larger when there are less screening charges in the atom. Interestingly and surprisingly, although there can be a great number of electronic configurations of a heavy atom in an ionized sample, with these configurations randomly distributed over those atoms in the sample, it should still be possible to carry out a form of phasing by anomalous diffraction at extreme doses [26]. Under these conditions, the molecular form factor will still exhibit Bijvoet differences, albeit reduced in magnitude. Making up for this reduction is a predicted bleaching of the heavy-atom scattering factors at these high intensities that increases the anomalous differences used in multi-wavelength anomalous diffraction—multi-wavelength anomalous diffraction (MAD) phasing (but note that a modified formalism must be employed). The atomic bleaching could be used for phasing by a method similar to single isomorphic replacement [55] or radiation-damage-induced phasing [56], by taking measurements at high and low doses at a photon energy above the neutral-atom absorption edge [27]. The scattering of the heavy atoms will be different at these two doses, whereas the rest of the structure remains largely invariant, except for an overall decrease in scattering strength. The normalized effective scattering factor of Fe is plotted in figure 6 as a function of pulse fluence, showing a change in scattering factor that is larger than the difference obtained when changing photon energy across the absorption edge. A similar behaviour is seen at for 6 keV photons (which is below the Fe absorption edge), albeit at higher dose. The dose for which the effective atomic scattering factor of Fe begins to bleach is about 0.5 GGy (4 × 10^11^ photons μm*^−^*^2^ at 8.1 keV). These calculations of the atomic bleaching do not take into account the electron cascades, which could certainly give rise to a greater number of core–shell ionizations than predicted here, possibly lowering the bleaching dose.

### (b) Effect of random ionization

The distribution of photoionized atoms will be random throughout the sample, although with an ‘occupancy’ that is weighted by the relative absorption cross sections of the various atomic species (giving a means to locate more rapidly ionizing atoms and hence new phasing methods as discussed in §4.1.). The distributions of atoms that are ionized by the electron cascades are centred on the primary absorption sites and depend on the Auger and photoelectron energies. For a sufficiently high dose that gives many photoionization sites per electron cascade volume, we can assume that the collisionally ionized atoms are also randomly distributed. For atomic species that ionize at roughly the same rate, there is no inherent length scale of the perturbation, because we assume all ionizations to be independent of each other. In this case, the effect of both the coherent and incoherent averaging over the experiment gives rise to diffraction that is the incoherent sum of two terms: the diffraction from the average crystal or particle and the diffraction from the difference structure from the average [57]4.3
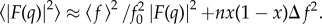


Here, we have made a simplification of a binary population consisting of *n* atoms of a single element that have a fraction *x* ionized; 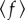 is the average scattering factor over this population, *f*_0_ the unionized scattering factor and Δ*f* the difference in scattering factors of the ionized and unionized atoms. More generally, the average crystal will have an overall reduction in scattering strength as per the average ionization of atoms, and this term will give rise to a uniform reduction of the strength of the diffraction, falling to zero if there are no more electrons left in the sample. The second term will give rise to diffuse scattering that will have no dependence on resolution or scattering vector *q* (because there is no associated length scale to the ionization occupancy) other than the fall off of the average atomic scattering factor, *f*(*q*). We must add to the diffraction signal the scattering from the ejected free electrons, if they remain trapped in the sample. Again, we consider these to be completely randomly distributed, giving rise to a uniform background proportional to the total number of free electrons. In the extreme case of a plasma where all electrons have been stripped off every atom, and most of these electrons are trapped, only this diffuse electron–gas diffraction will remain. The total number of scattered photons from this state will be approximately equal to the scattered photons from the undamaged sample, because there are the same number of electrons in both cases. For crystals, however, it is easy to distinguish these extremes, because the undamaged crystal confines and concentrates the scattered photons into Bragg peaks. Thus, the contribution from the average-ordered crystal can be extracted from the diffuse scattering by a simple background subtraction around each Bragg peak. This is not the case for single-particle diffraction, where the subtraction of a uniform offset could be carried out after data assembly so as to maximize the diffraction contrast.

The measured diffraction pattern is integrated over the course of the pulse, and as the degree of ionization builds up during the pulse the contribution to the diffraction pattern will decrease by a factor 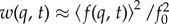, which depends on the average degree of ionization that is plotted in figure 3 [45]. For crystals and intense pulses, this will lead to a gating of the diffraction pattern, where Bragg diffraction will end when all atoms are completely ionized. From figure 3, it can be seen that this occurs after about 3 fs for a dose rate of 60 GGy fs*^−^*^1^ (i.e. a dose of about 200 GGy) and after 60 fs at a rate of 0.6 GGy fs*^−^*^1^ (40 GGy dose), giving a weak dose-rate dependence on total achievable dose of approximately 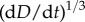. (The time that the sample becomes transparent owing to ionization varies approximately as 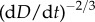. We note that even at lower doses than those to fully ionize atoms, the ionization acts to weight the diffraction in favour of the start of the pulse. Diffuse scattering will increase over the duration of the pulse, and the measured pattern will consist of the Bragg component (primarily from the start of the pulse) and a diffuse component (primarily from the end of the pulse). The peaks can be distinguished and separated from the diffuse, effectively shortening the pulse duration, for all but the smallest nanocrystals. When the width of the Bragg peak is dominated by the nanocrystal shape, the ratio of counts per unit detector solid angle in the diffuse background compared with Bragg counts depends on the ratio of the area of a Bragg peak to the area of the reciprocal unit cell, or 1/*N*^2^, where *N* is the number of unit cells in width of the crystal.

### (c) Effect of random atomic displacements

**Figure 7.**
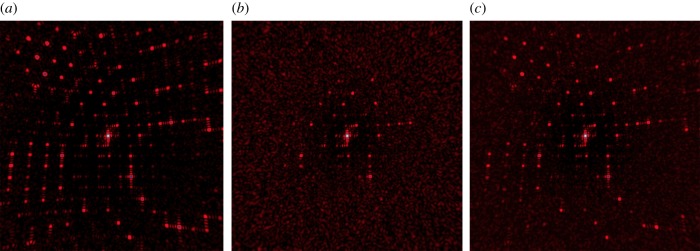
Simulated diffraction patterns of a 5 × 5 × 5 unit cell cubic crystal undergoing an X-ray induced explosion, following the dynamics shown in figure 5. (*a*) The undamaged pattern simulated with no atomic displacements; (*b*) the pattern simulated with 1 Å RMS atomic displacement and (*c*) the pulse-integrated pattern where the RMS displacement reached 1 Å at the end of the pulse. The unit cell length is 25 Å. (Online version in colour.)

The effect on the diffraction pattern of random and isotropic atomic motion induced by the pulse can be calculated through the usual Debye–Waller formalism for such disorder. Here, too, the diffraction pattern is given as a sum of the Bragg component (calculated as the diffraction of the static structure of the mean atomic positions) and a diffuse component [57]:
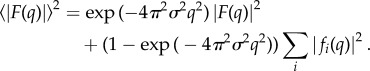
As opposed to random ionization occupation, there is a very definite length scale of the atomic displacement, which gives a *q*-dependent term that acts as a low-pass filter at a resolution of 2*πσ* and gives rise to increased diffuse scattering above that resolution. (The presence of the 2*π* can be understood from the fact that atomic displacements lead to changes in the phases of the waves scattered from these atoms, such that the interference at the Bragg peaks is no longer constructive. This result also holds for incoherent addition of single-particle patterns.) At the beginning of the pulse, the X-ray-induced atomic displacements are zero so at that point only Bragg diffraction contributes, plus any diffuse scattering owing to inherent disorder of the unexposed crystal. As the pulse progresses, the low-pass filter cut-off moves to lower resolution according to the evolution of *σ* as shown in figure 5. Thus, there will be a particular time during the pulse when the filters cut-off is reached for a particular resolution, which we refer to the turn-off time [12]. We can think of the pulse-integrated effect again as a gating effect where, for a particular resolution, some initial fraction of the pulse (given by the ratio of the cut-off time to the pulse duration) contributes. To illustrate the effect of disorder on the diffraction pattern, we simulate the diffraction pattern of a 5 × 5 × 5 unit cell crystal undergoing disorder as given in figure 5, using equations (4.1) and (4.2). The simulations, shown in figure 7 do not include any photon noise, and show the instantaneous diffraction pattern achieved with no atomic motion, an RMS displacement of *σ* = 1 Å (achieved at the end of the pulse), and the pulse-integrated pattern. It is seen that although at the end of the pulse the highest-resolution peaks no longer contribute to the pattern, the pulse-integrated pattern still shows those peaks.

The turn-off time occurs earlier for higher resolutions and progresses to low resolutions, giving a pulse-integrated filtering function that is not Gaussian but, at resolutions above about 20 Å, approximately follows an inverse power law of *q* [12]. In addition, the data will be modulated by a Gaussian term owing to the initial inherent disorder of the unexposed sample, and at low doses, this usual behaviour will dominate the Wilson plot of the dataset.

The diffuse scattering owing to the evolving disorder begins first at high resolution and develops in to lower resolution as the pulse progresses. The pulse-integrated diffuse scattering follows the complement of the filtering function, because the loss in Bragg scattering at a particular resolution is directed into diffuse scattering.

The combination of the disordering owing to ionization and atomic displacements leads to the contribution to Bragg diffraction, over the pulse of duration *T*, according to [45]4.5
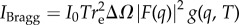
and4.6
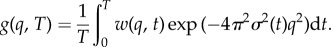
It is seen that the Bragg counts are modulated by a dimensionless factor *g*(*q*, *T*), referred to as the dynamic disorder function [12]. This factor gives the proportion of the pulse that contributes to Bragg diffraction, gated by the RMS atomic displacement *σ* exceeding 2*π*/*q* or the degree of ionization *w* dropping to zero.

**Figure 8.**
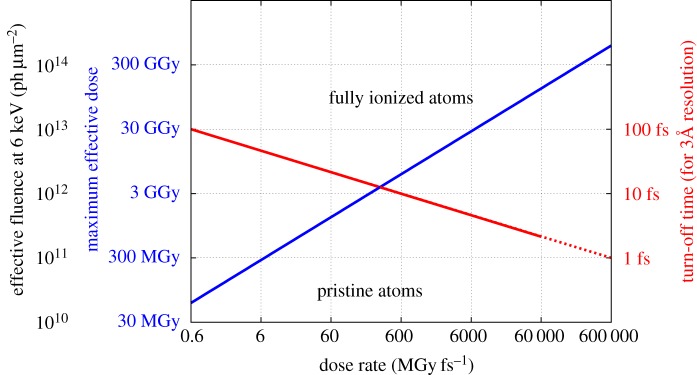
The effective fluence (or total diffracted signal) that can be achieved in a single pulse is a product of the pulse intensity (or equivalently dose rate) and the time the sample diffracts (the turn-off time). The turn-off time decreases with increasing dose rate, but the result is an increase in total achievable signal. (Online version in colour.)

For dose rates below about 100 MGy fs*^−^*^1^, the ionization rate is low enough that it is the atomic displacement that gates the diffraction. As the dose rate increases to the point that atoms become completely stripped during the pulse, the gating occurs owing to that ionization before even the onset of atomic motion. Considering first the gating owing to atomic displacements, we see from figure 5 that as the dose rate is increased the turn-off time also decreases, giving less time for the contribution to Bragg diffraction. The turn-off time varies approximately with 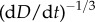, and hence in that reduced time, the increase in the number of photons per second from that higher dose rate more than compensates for the reduced time. The dose rate multiplied by the turn-off time gives a maximum effective dose that can be achieved at a given resolution and given dose rate, plotted in figure 8. This maximum effective exposure increases with increased dose rate, with the two/thirds power of dose rate. Thus, our model suggests that highest possible diffraction signals (or measurable diffraction from the smallest possible samples) are achieved with highest dose rate, up to the point of completely ionizing every atom that would occur at approximately 20–200 GGy depending on dose rate and if the photoelectron cascades can be avoided (by isolated samples or short times). Increased dose rate is achieved by increasing the X-ray intensity which can be done either by focusing the beam to a smaller spot, compressing the pulse in time or increasing the peak power of the source. The LCLS, for example, routinely produces 100 GW pulses, and schemes for exceeding 1 TW have been proposed [58].

### (d) Effect of correlated structural changes

The different rates of ionizations of various atomic species in the sample, owing to their different photoabsorption and electron collision cross sections, must give rise to a component of the structural evolution during the pulse that is not random or isotropic. The heavier elements certainly will ionize faster than lighter elements, initially creating an inhomogeneous distribution of positive charges. One predicted form of correlated motion that could take place during the pulse is the repulsion of light atoms from a rapidly ionizing heavy atom [24]. Any correlated motion of atoms that does occur will contribute to Bragg peaks and be measured in the final ensemble-averaged structure factors. The effect will be a blurring of electron density that may give rise to displacements of mean atomic positions and an effective increase in atomic *B* factors. An acceleration of an atom, as predicted by Jurek & Faigel [24], will not properly be represented by a harmonic motion of a *B* factor, however. It will depend on the pulse-weighted distribution of positions along the trajectory. One complicating factor is that, owing to Bragg termination caused by the uncorrelated atomic motion of the forming plasma, higher resolution Bragg peaks are gated at earlier times than at lower resolution. The turn-off times progress from high resolution to low, and hence the correlated motion will appear differently at different resolutions. Strictly, there will be no electron density that could give rise to the observed structure factors, but the result will approximately further blur the motion by the low-pass filtering effect of Bragg termination. However, to be observable, the correlated motion must outrun the Bragg gating, so that its contribution to the pattern adds coherently with that of the rest of the structure. It must move by a distance corresponding to the resolution of a Bragg peak in a time before that Bragg peak turns off. The displacement of the correlated motion must therefore be larger than 2*πσ*, meaning that it must be about six times faster than the diffusive motion. In the study of Jurek & Faigel [24], with a dose rate of 22.6 GGy fs*^−^*^1^ (10^13^ 12 keV photons per 10 fs per (100 nm)^2^) and after 10 fs, the Fe to nearest-neighbour C distance changes from 2.5 to 3.5 Å (with a standard deviation of about 0.5 Å), and the RMS displacement of the other C atoms is about 0.5 Å. That is, 10 fs corresponds to the time that the 3 Å resolution Bragg peak terminates. (This is longer than predicted by the simulations shown in figure 5, because photoelectrons can escape the 40 Å diameter cluster.) Further taking into account the acceleration of the nearest-neighbour C atoms away from the Fe ion, giving greater weighting to smaller displacements of these atoms during the pulse, this correlated motion (as modelled) would not be readily observable in a crystal diffraction experiment.

The largest and most observable correlated structural changes that occur during the pulse will be the ionization of atoms, changing the scattering atomic factors. The differences in ionization rates of various atomic species is quite apparent from their cross sections, as can be seen in figure 6. The effect of this will be that the electron density around heavy atoms will be misinterpreted, which could possibly lead to the wrong identification of atoms. Hau-Riege *et al*. [59] have shown that if the stoichiometry is known then the effect of varying ionization rates can be corrected directly in the data, for both single-particle and crystal data. Most interestingly, the differing ionization rates can lead to a means to determine the locations of a particular atomic species, to assist in phasing [27,32].

## 5. Optimum wavelength for X-ray free-electron laser diffraction

In crystallography using synchrotron radiation, the optimal photon energy is that which gives the greatest signal for the least radiation damage, favouring data collection at high photon energies of around 25–40 keV [60] depending on crystal size [61]. This condition maximizes the ratio of the effective scattering cross section to the effective absorption cross section, which only has a weak dependence on photon energy. Higher photon energies also beget higher energy photoelectrons that have longer mean-free paths. With high-intensity X-ray FEL pulses, there is no need to minimize the dose if the pulse can outrun the damage. The diffraction from small or weakly diffracting crystals can be increased by reducing photon energy since, for a given crystal, the total number of photons in a fully recorded Bragg peak increases with *λ*^3^ for a given number of incident photons per unit area [61] (note that the *λ*^3^ is obtained by counting within volumes of *q* space and that average scattered counts per Bragg peak per pattern scale as *λ*^2^, but the integration achieved by the Monte Carlo approach, for example, gives an additional factor proportional to *λ*. Another way to think of this is that the probability of observing a particular Bragg peak in a still pattern scales with *λ*). The incident number of photons for a given pulse energy or intensity scales with *λ*. As wavelength is increased, the dose increases, which causes the turn-off time of Bragg peaks to shorten, as illustrated in figure 5. The RMS displacement, plotted in figure 5, roughly increases with the one-third power of dose rate, and the turn-off time inversely scales with the one-fourth power of dose rate. Dose is proportional to the photoabsorption cross section (equation (2.1)) which is proportional to *λ*^3^, away from absorption edges, and hence the turn-off time scales as *λ^−^*^3/4^. The overall signal (in total scattered photons, for a noise-free detector) is thus proportional to the product of the number of available photons in an FEL pulse (*λ*), the average scattered photons per incident photon fluence (*λ*^3^) and the diffraction gating time (*λ^−^*^3/4^), giving a result that scales as *λ*^13/4^, or approximately *λ*^3^. Increasing wavelength has its cost of limiting the spatial resolution, which could be optimized by detecting as large scattering angles as possible, to a limit of *λ*/2 for complete backscattering. In this dose-rate regime where atomic motion gates the pulse, the analysis suggests that highest signals are achieved by working at the longest wavelength that supports recording diffraction at the resolution of interest. A photon energy of 4 keV could give almost 10 times more Bragg photons than 8 keV.

In large samples where photoelectrons are trapped and at high-dose rates exceeding about 1 GGy fs*^−^*^1^, the gating is dominated by ionization, and the time to reach scattering transparency varies inversely with the two-third power of dose rate. Following the same reasoning as above, the turn-off time in this regime scales as *λ^−^*^2^, giving an overall signal that varies as *λ*^2^. For the ‘average protein’, the dose rate of 1 GGy fs*^−^*^1^ corresponds to an intensity of 4 × 10^11^ photons μm*^−^*^2^ fs*^−^*^1^ or 4 × 10^19^ W cm*^−^*^2^ at 6 keV photon energy, or 400 GW of delivered source power focused into a 1 μm^2^ spot.

## 6. Conclusion

Serial crystallography makes use of two distinct strategies to measure high-resolution structure factors of macromolecular crystals free of radiation damage. The most obvious is that the short pulses outrun the radiation damage processes, allowing doses thousands of times higher than tolerable when using synchrotron sources. A focused high-intensity pulse from an X-ray FEL completely vaporizes the sample, but gives rise to a flash snapshot diffraction pattern before damage occurs. The higher achievable dose translates into a stronger diffraction pattern for a given crystal size, allowing for data collection from crystal sizes that can be only tens of unit cells in width. To use the one-crystal one-pulse approach afforded by X-ray FEL pulses, several other new and innovative technologies were combined with the FEL source itself: area detectors with frame rates to match the FEL pulse rate [10], and methods to deliver the sample at that rate to the focused X-ray beam [62]. This combination allows millions of diffraction patterns to be recorded in an experiment, and opened up the second way to improve signals from limited-size crystals by indexing of individual patterns followed by integration over many patterns to build up signal and average away the noise in the background.

In this review, we outlined radiation damage processes that can occur during and soon after the duration of the pulse. The degree of damage per elastically scattered photon (contributing to the diffraction pattern) is rather unfavourable for X-rays, with about 30 photoionizations occurring per scattered photon, and each of those photoionizations giving rise to a cascade of over 100 electron-induced ionizations. Less damage occurs during an exposure if high-energy photoelectrons can escape the sample instead of setting off an electron cascade, if the beam size is smaller than the range of the photoelectrons, or if the pulse duration is significantly less than the ≈100 fs duration of the cascade. A population of hollow atoms can build up if the core–shell ionization rate exceeds the Auger decay rate, leading to a frustration of absorption for only a small decrease in scattering strength.

Obviously, only time during the pulse contributes to the diffraction pattern, and diffraction contributions are weighted to earlier times in the pulse owing to a gating of Bragg peaks by uncorrelated atomic displacements and, at highest doses, owing to a loss of scattering strength of ionized atoms. From plasma physics simulations, the rate of diffusion of atoms depends on dose rate, whereas the degree of ionization depends predominantly on dose. We identified several dose regimes, including the single-electron ionization per atom at 400 MGy, below which scattering predominantly occurs from unionized (or pristine) atoms. Single core–shell photoabsorption saturates when the number of photons per unit area is equal to the inverse of the absorption cross section, giving a dose for a particular element equal to the photon energy multiplied by the number of atoms per unit mass. For 6 keV photon energy, this corresponds to 50 GGy for carbon, which is comparable to the dose to strip every electron from every element (40 GGy at a dose rate of about 1 GGy fs*^−^*^1^) if the electron cascades can fully develop. The one-photon-per-atom dose is lower for heavier elements, leading to an inhomogeneity in ionization that can be used as a method for phasing [27,32]. At dose rates faster than about 1 GGy fs*^−^*^1^, Bragg termination occurs within 10 fs or less. Effective doses above 10 GGy could be achieved at higher dose rates. For example at 100 GGy fs*^−^*^1^, the estimated Bragg termination time is 1 fs, which is shorter than the cascade generation, giving an effective exposure of 100 GGy, over 3000 times the tolerable limit for cryogenic samples in ‘slow’ exposures. This factor indicates how much smaller in volume crystals can be compared with synchrotron measurements, but we note that additional reduction factors in crystal size are achieved in serial crystallography by forgoing the measurement of fully integrated peaks from each crystal and from the benefit of averaging over many crystals. These latter aspects of serial crystallography can also be applied to measurements using synchrotron radiation [63,64], and are crucial for applying the method to smaller and smaller crystals, all the way to the single molecule.
